# A Final Voyage Framed by Balance and Contrast

**DOI:** 10.3201/eid2701.AC2701

**Published:** 2021-01

**Authors:** Byron Breedlove

**Affiliations:** Centers for Disease Control and Prevention, Atlanta, Georgia, USA

**Keywords:** art science connection, emerging infectious diseases, art and medicine, about the cover, a final voyage framed by balance and contrast, the fighting Temeraire (tugged to her last berth to be broken up), public health, waterborne infections, water safety, diarrheal diseases, cholera, schistosomiasis, Guinea worm disease, giardiasis, dysentery, Legionnaires’ disease

**Figure:**
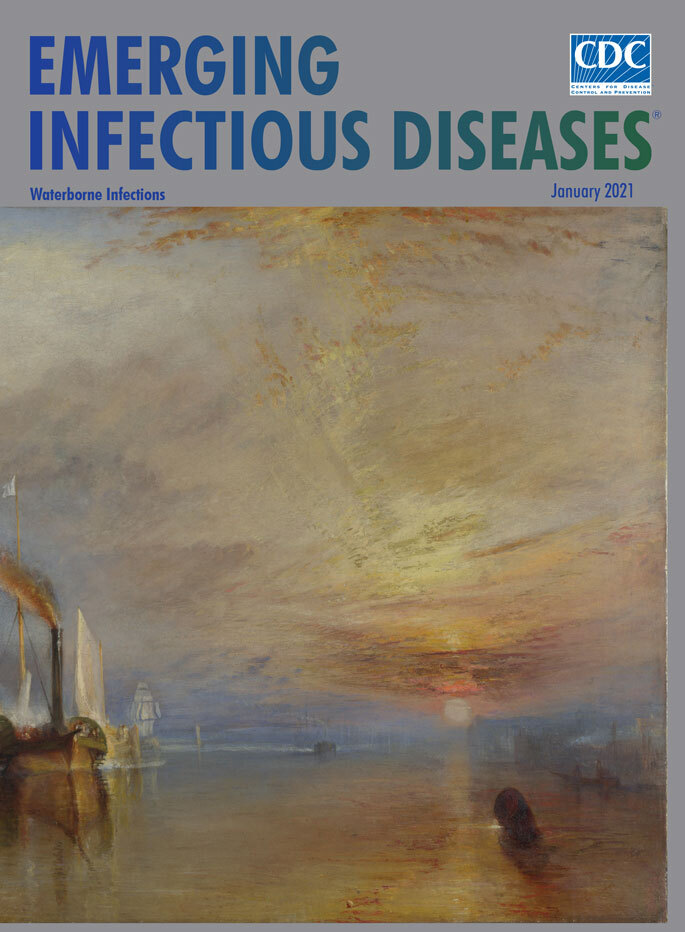
**Joseph Mallord William Turner (1775–1851). The Fighting Temeraire (Tugged to Her Last Berth to Be Broken Up), *1838*.** Oil on canvas, 36 in × 48 in/91cm × 122 cm. National Gallery, London, UK. Public Domain.

Considered among Britain’s greatest artists, *Joseph Mallord William Turner* (known better as J. M.W. Turner) was compelled by a lifelong need to create; he is known to have completed more than 550 oil paintings, 2,000 watercolors, and 30,000 works on paper. The National Gallery in London calls Turner “the painter of light” because of his prominent use of brilliant colors in landscapes and seascapes. Water, both turbulent and tranquil, appears in many of his better-known works, and a measure of irony is that he later died from an illness caused by contaminated water. 

Art critic Jonathan Jones ventures the opinion that “One measure is the fascination an artist holds, not just for the general public, but for other artists. If an artist of the past is still haunting, provoking and inspiring modern artists, that has to suggest some deep vitality. To this day, Turner haunts art in that way. It is not yet done with the grandiose after-echoes of his smoky light.” 

Before admission to the Royal Academy of Arts when he was 14 years old, Turner had received little formal education. Soon acknowledged as a prodigy, Turner became the youngest painter to have works featured in the Academy’s annual exhibition, and at age 26, was the youngest student to be designated Royal Academician, the Academy’s highest rank. He also gained experience working with architects and draughtsmen and painting theatre sets. 

According to art historian Elizabeth Barker, Turner was “an innovator who has been hailed as a forerunner of modernist abstraction.” She explains how he honed his method on a series of small topographical watercolors: “To create details, Turner scraped, blotted, and wiped the paint while it was still wet, and scratched into or drew on dry surfaces.” Turner also applied that technique to oil painting. Barker notes, “He built up from foundations of color to create uniquely evocative shapes and glowing forms.” 

In the final phase of his career, “Turner’s painting became more vigorous, intense and liberated than ever before.” states art critic Alastair Sooke. Turner is remembered not only for those colorful, luminous works but also for the range of subjects portrayed in his art. Some works depicted tragic and horrific events such as wars, fires, and shipwrecks; others offered insightful commentaries on the contemporary world’s transformation as the industrial revolution gained traction. 

Turner completed* The Fighting Temeraire*, this month’s cover image, in 1838, at the zenith of his career, and the painting was displayed at the Royal Academy in 1839. Launched in 1798, the UK Navy ship HMS *Temeraire* saw naval action in 1805 during the Battle of Trafalgar, when it rescued Admiral Horatio Nelson’s besieged flag ship HMS *Victory* and captured two French warships. After being repaired, the *Temeraire* blockaded French fleets and supported British military operations off the coast of Spain. Historical records indicate that in 1811 an outbreak of yellow fever swept through the *Temeraire, *killing nearly 100 of the ship’s crew members, double the number who died at Trafalgar. 

Later moored at the Sheerness Dockyard, UK, the warship served as a floating prison, barracks for new recruits, depot ship, and stationary guard ship. Per standard orders, the ship’s cannons, masts, and rigging would have then been removed for reuse. 

For much of his life, Turner lived close to the Thames, though no evidence suggests he saw the man-of-war’s final voyage, and he took substantial artistic license in his romanticized depiction. Turner restored the masts and sails to the pallid titan as a nod to its glory days. He framed the *Temeraire *with a triangle of blue sky, the massive vessel drifting high in the water toward the center of the painting, so that the heroic ship looms over the squat tug dragging it to its destruction. 

Turner expertly draws on balance and contrast in the painting. Behind the ghostly lit *Temeraire*, a square-rigged sailing vessel and other faintly visible ships head down the river toward the setting sun. Lit by the sun, the stout tug, rendered in bold colors, churns upstream and bellows smoke across the derelict’s spar where a flag would have waved. Vivid warm colors play across the low clouds and reflect on the water’s surface, calling attention to the low sun. In contrast, a faint crescent moon wreathed in cool blues and grays hangs near the upper left of the canvas and contrasts with the brilliant sunset. 

Turner’s choice of colors and positioning the tug in front of the *Temeraire* serve both as an elegiac farewell to the waning dominance of wooden sailing vessels and an acknowledgment the growing reliance on steam-powered ships. The quiet steadiness of wind power is yielding to the loud urgency of steam engines.

Turner was an eccentric, reclusive character, especially in his later years. A cholera epidemic in 1831–32 had terrified him, and some 20 years later, when he was already in declining health, a severe case of this waterborne disease led to his death. 

Today, diarrheal diseases, including cholera, kill more children than AIDS, malaria, and measles combined. According to the Centers for Disease Control and Prevention, 780 million people lack access to safe water and 2.5 billion people lack access to adequate sanitation. Such conditions also contribute to the spread of other waterborne diseases such as schistosomiasis, Guinea worm disease, giardiasis, and dysentery. 

Access to piped water does not guarantee freedom from the threat of waterborne illness. Legionnaires’ disease is caused by *Legionella *bacteria that can grow and spread in poorly maintained plumbing, especially in buildings. Collier et al. found that during 2000–2015, a total of 7.15 million waterborne illnesses occurred each year, resulting in 601,000 visits to emergency departments, 118,000 hospitalizations, and 6,630 deaths annually. The challenges of providing access to safe water and addressing pathogens that live in manufactured water systems underscore the need for more public health resources to address waterborne infections.
